# Stratifying dementia risk factors: A prediction model and hypothesis‐driven analysis

**DOI:** 10.1002/alz.70870

**Published:** 2025-10-28

**Authors:** Daniel Arnold, Rodrigo C Barros, João Pedro Ferrari‐Souza, Marco Antonio de Bastiani, Eduardo R Zimmer, Wyllians Vendramini Borelli

**Affiliations:** ^1^ Graduate Program in Biological Sciences: Pharmacology and Therapeutics Universidade Federal do Rio Grande do Sul (UFRGS) Porto Alegre Rio Grande do Sul Brazil; ^2^ Graduate Program in Computer Science (PPGCC) School of Technology Pontifical Catholic University of Rio Grande do Sul (PUCRS) Porto Alegre Rio Grande do Sul Brazil; ^3^ Graduate Program in Biological Sciences: Biochemistry Universidade Federal do Rio Grande do Sul (UFRGS) Porto Alegre Rio Grande do Sul Brazil; ^4^ Department of Pharmacology Universidade Federal do Rio Grande do Sul (UFRGS) Porto Alegre Rio Grande do Sul Brazil; ^5^ The McGill Centre for Studies in Aging McGill University Montreal Quebec Canada; ^6^ Brain Institute of Rio Grande do Sul Pontifical Catholic University of Rio Grande do Sul (PUCRS) Porto Alegre Rio Grande do Sul Brazil; ^7^ Department of Morphological Sciences Universidade Federal do Rio Grande do Sul (UFRGS) Porto Alegre Rio Grande do Sul Brazil; ^8^ Memory Center Hospital Moinhos de Vento Porto Alegre Rio Grande do Sul Brazil

**Keywords:** Alzheimer's disease, cognitive impairment, machine learning, prognosis, SHAP

## Abstract

**INTRODUCTION:**

Most older adults present multimorbidity, but dementia risk factors are typically analyzed individually. Direct methodological comparisons evaluating simultaneous multiple risk factors are essential to provide the realistic effects of multimorbidity. We aimed to compare hypothesis‐ and data‐driven approaches for dementia risk stratification in a real‐world cohort.

**METHODS:**

We analyzed 9606 participants from the National Alzheimer's Coordinating Center (NACC) Uniform Data Set (2005–2023) using machine learning with interpretability analysis and survival models to simultaneously evaluate 13 risk factors for incident dementia conversion.

**RESULTS:**

A total of 877 participants (9%) developed dementia over (mean ± SD, 6 ± 4.2) years of follow‐up. Both approaches consistently identified four key predictors: age, depression, low education, and body mass index (protective). Convergent findings across methodologies demonstrated robust factor identification despite different analytical paradigms.

**DISCUSSION:**

In this direct methodological comparison, age, depression, and low education emerge as major dementia risk factors regardless of analytical approach. Convergent interpretability of these approaches support simultaneous multifactorial risk assessment in clinical practice.

**Highlights:**

- Data‐ and hypothesis‐driven approaches identified convergent key risk factors
- Age, depression, and low education are major risk factors for dementia
- Higher body mass index was unexpectedly protective against dementia conversion
- Multimorbidity requires simultaneous evaluation of multiple risk factors
- Real‐world analysis reveals complex interactions between dementia risks

## 1 BACKGROUND

Dementia represents the final stage of a prolonged pathological process that ultimately leads to loss of functional independence. The global burden of dementia presents a significant public health challenge, affecting over 47 million people worldwide with economic costs exceeding US$1 trillion.^1^ Although novel disease‐modifying therapies have recently been approved for treating Alzheimer's disease (AD), the most common cause of dementia, their clinical benefits remain limited.^2^ In the absence of cost‐effective treatments for dementia and its underlying causes, prevention strategies represent a promising avenue for transforming the global dementia landscape.^3^

Given the constraints of public health budgets, the strategic prioritization of dementia risk factor control has become essential. Identifying the most impactful modifiable risk factors represents a critical public health strategy, one that is supported by demonstrated cost‐effectiveness and reinforced by international collaborative efforts.^3^ Globally, nearly half of all dementia cases globally can be attributed to 14 modifiable risk factors.^4^ However, the prevalence profile of these risk factors demonstrates considerable regional variation across the globe, highlighting that regional analysis is pivotal for public health strategies in specific populations.^5^ In the United States, hypertension has been identified as the most significant preventable risk factor for dementia,^6^ whereas in Brazil, low educational attainment and depression have emerged as the most impactful modifiable risk factors.^7^ Therefore, stratifying the most prevalent risk factors of dementia has become a global research priority.^8^

The relationship between risk factors and dementia has been investigated using diverse methodological approaches. In older adults, modifiable risk factors for dementia are often studied individually rather than in combination.^4^ However, more than half of older adults present with multimorbidity in clinical practice.^9^, ^10^ Examining risk factors in isolation may inadequately capture the complex interactions between multiple coexisting conditions. The relative risk of hypertension for incident dementia is typically evaluated as an independent variable,^4^ a method that overlooks its high comorbidity with conditions like hypercholesterolemia and other dementia risk factors.^11^

Consequently, there is growing interest in strategies that can mitigate analytical biases while enhancing the predictive capacity of clinical data using risk factors of dementia. Data‐driven models offer a unique opportunity to simultaneously evaluate multiple dementia risk factors and their interactions.^12^ Machine learning (ML) techniques have recently demonstrated high accuracy in both identifying dementia diagnoses and predicting risk across various clinical and research settings.^12^, ^13^ These data‐driven ML approaches may be particularly valuable for capturing complex interactions between risk factors in adults with multimorbidity, providing complementary insights to traditional hypothesis‐driven analytical frameworks.

Despite the growing application of both hypothesis‐driven and data‐driven approaches in dementia research, direct methodological comparisons using identical datasets and standardized risk factor definitions remain limited in evaluating multimorbidity. Previous studies have focused primarily on prediction performance optimization^12^ or employed broader feature sets including biomarkers,^14^ rather than systematically comparing how different analytical paradigms interpret the same clinical variables simultaneously. Furthermore, most investigations have not concentrated specifically on cognitively unimpaired populations at baseline, despite the critical importance of early risk stratification for preventive interventions. The lack of direct comparisons between traditional survival analysis and modern ML approaches, using clinically established risk factors simultaneously, represents a significant gap in understanding how these methodologies complement each other in assessing dementia risk in the context of multimorbidity.

Here we aimed to stratify risk factors for incident dementia by examining both modifiable and non‐modifiable factors in cognitively unimpaired individuals. We employed both hypothesis‐driven and data‐driven approaches on the same longitudinal dataset from real‐world memory clinics to provide a comprehensive methodological comparison.

## 2 METHODS

### 2.1 Study sample

Data were derived from the National Alzheimer's Coordinating Center (NACC) Uniform Data Set (UDS).^15^, ^16^ The UDS comprises prospective cohort data from the Alzheimer's Disease Research Center (ADRC) program of the National Institute on Aging, aimed at facilitating multicenter collaborative research on AD and other neurodegenerative disorders. This dataset includes memory clinic information gathered from 2005 to 2023 across 46 ADRCs in the United States. It encompasses the sociodemographic characteristics of participants and their companions, medical history, behavioral symptoms, and cognitive and functional status. The clinical dementia diagnosis made by each center was based on established clinical diagnostic criteria derived from the standardized UDS clinical evaluation. Comprehensive details regarding the diagnostic criteria utilized in the UDS protocol and related guidance have been documented in previous publications.^17^ The NACC study obtained ethical approval from the institutional review board at each participating site before data collection, and all participants provided written informed consent. This study was classified as exempt from institutional ethical approval, since it used previously collected de‐identified data. This analysis adheres when possible to the reporting guidelines set forth by the Transparent Reporting of a Multivariable Prediction Model for Individual Prognosis or Diagnosis (TRIPOD).^18^ Data were retrieved in November 2024.

### 2.2 Outcome variables

The primary outcome measure was the transition from cognitive normality at baseline to a stable all‐cause dementia diagnosis during the longitudinal follow‐up period. To enhance diagnostic precision and mitigate potential diagnostic instability inherent in longitudinal studies, rigorous diagnostic criteria were implemented in this study. Participants were classified as having converted to dementia only if they: (1) received an initial dementia diagnosis, and (2) maintained this diagnosis consistently across later assessment points. Conversely, the cognitively unimpaired cohort comprised participants who remained without a dementia diagnosis in the later visits.

**RESEARCH IN CONTEXT**

1. **Systematic review**: Existing literature was reviewed in Medline for studies evaluating modifiable and non‐modifiable risk factors of dementia. Although many risk factors of dementia have been identified, there is a lack of studies evaluating multiple risk factors simultaneously using real‐world longitudinal studies.
2. **Interpretation**: We conducted data‐ and hypothesis‐driven approaches stratifying 13 risk factors of dementia (11 modifiable, 2 non‐modifiable) using a longitudinal, nationwide dataset (National Alzheimer's Coordinating Center [NACC]) to identify the most impactful risk factors of conversion of cognitively unimpaired individuals to dementia. Age, depression, and low education were consistently identified in both models analyzed, whereas high body mass index was protective.
3. **Future directions**: Evaluating multiple risk factors of dementia simultaneously using two analyses models stratified risk factors differently. Hypothesis and data‐driven approaches should be conducted to capture the complexity of risk factors of dementia. The next studies should update the current analysis with newly identified risk factors.

Participants with diagnostic trajectory inconsistencies were excluded in hypothesis‐ and data‐driven analyses, specifically those who experienced diagnostic regression from dementia or mild cognitive impairment (MCI) and presented a history of major neuropsychiatric disorders. The detailed fluxogram of the criteria to build the cohort is shown in Figure S1.

Diagnostic classifications were uniformly determined at each NACC site, adhering to established international diagnostic criteria for each dementia subtype as AD,^19^ vascular dementia,^20^ Lewy body dementia (LBD),^21^ and frontotemporal dementia.^22^

### 2.3 Risk factors evaluated

We evaluated well‐established modifiable risk factors for dementia described by the Lancet Commission,^4^ along with two non‐modifiable factors (age and sex). Of the 14 modifiable risk factors, 11 were identified in the NACC dataset: less education, hearing loss, hyperlipidemia, depression, traumatic brain injury (TBI), diabetes, smoking, hypertension, obesity, excessive alcohol use, and visual loss. Physical inactivity, air pollution, and social isolation were unavailable in the NACC UDS.

Composites of the modifiable and non‐modifiable risk factors for dementia were constructed using various fields to maximize the use of available data. Specifically, obesity was assessed using the body mass index (BMI), and depression was evaluated according to the Geriatric Depression Scale (GDS). We defined obesity as a BMI higher or equal to 30,^23^ depression as a GDS higher than 4,^24^ and low education as lower or equal to 8 years.^25^ Baseline demographic and clinical characteristics were assessed at study entry, including age, educational attainment, BMI, depression status, hearing impairment, elevated low‐density lipoprotein (LDL) cholesterol, history of traumatic brain injury, diabetes, smoking history, hypertension, excessive alcohol consumption, and visual impairment. The analysis incorporated only participants with complete data for these variables.

### 2.4 Data‐driven approach

**FIGURE 1:**
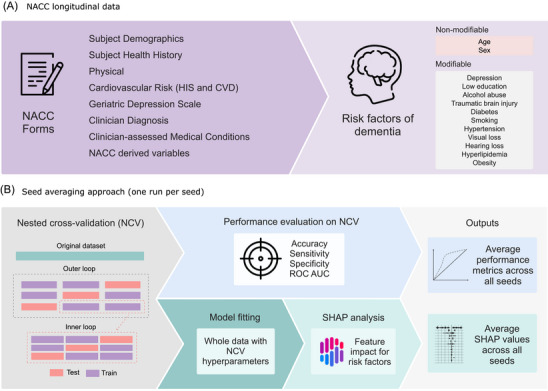
Two main stages of the data‐driven approach: (A) Using the NACC longitudinal data, Uniform Data Set forms were evaluated for 13 risk factors, categorized as non‐modifiable (age, sex) and modifiable (depression, low education, and so on). (B) The seed averaging approach included a NCV approach, followed by a performance evaluation of NCV, model fitting, SHAP analysis, and the final model trained on the full dataset with the optimized hyperparameters. CVD, cerebrovascular disease; HIS, Hachinski Ischemic Score; NACC, National Alzheimer's Coordinating Center; NCV, nested cross‐validation; SHAP, SHapley Additive exPlanations.

The ML approach was developed and evaluated following the pipeline depicted in Figure 1A,B. To predict the conversion to dementia from our set of risk factors, we selected the XGBoost (Extreme Gradient Boosting) algorithm.^26^ XGBoost is a highly efficient and scalable implementation of the gradient boosting framework, which builds a predictive model in the form of an ensemble of weak decision trees. It is a sophisticated algorithm that fits subsequent trees to the residual errors of the previous ones, effectively creating a powerful and robust classifier.

Although a traditional model like multiple logistic regression could be used for this classification task, XGBoost was used due to several key advantages. First, unlike regression models that assume a linear relationship between predictors and the outcome, XGBoost can intrinsically capture complex, non‐linear relationships and high‐order interactions among risk factors without requiring them to be manually specified. Second, its built‐in regularization techniques (L1 and L2) help to prevent overfitting, which is crucial when dealing with a large number of predictors. Finally, XGBoost's superior performance has been demonstrated consistently in a wide array of applications and ML competitions involving structured (tabular) data, often outperforming other algorithms such as random forests and support vector machines.^27^ The model's complexity is managed through the tuning of hyperparameters, which was implemented by a hyperparameter optimization (HPO) framework,^28^ with the search space and its impacts described in Table S1; to ensure a robust evaluation of the model's performance and minimize the risk of overfitting, a nested cross‐validation (NCV) approach was implemented, using a proportion of one third to test to two thirds of training and validation,^29^ with HPO occurring in the inner loop of the NCV. Following the training and optimization of the model, its performance was assessed in the outer loop of the NCV, providing an unbiased estimate, using accuracy, sensitivity, specificity, the area under the receiver‐operating characteristic (ROC) curve (ROC AUC) and the area under the precision‐recall curve (PR AUC). The accuracy, sensitivity, and specificity evaluated in the probability threshold defined by the maximum value of Youden's J index, which is defined as (sensitivity + specificity − 1), was used to determine the optimal cutoff point. This threshold is applied to the model's predicted probability score for each participant. A participant's score above this threshold leads to a classification of “high‐risk for dementia,” whereas a score below it results in a “low‐risk” classification. This method identifies the probability value that best maximizes the balance between the true positive and true negative rates. In addition to the HPO and NCV strategies to mitigate overfitting, we also compared and evaluated the metrics of ROC AUC and PR AUC on the training and validation sets in the inner loop of the NCV to analyze the overall fitting.

To provide insights into the model's decision‐making process and build trust in its predictions, the SHAP (SHapley Additive exPlanations) framework was employed. SHAP values are a game‐theoretic approach to explaining model predictions by assigning each feature an importance value for a particular prediction.^30^ SHAP values offer a unified and theoretically sound way to understand the contribution of each feature to the model's output. This analysis helps identify the key factors influencing the model's predictions, thereby enhancing the interpretability and transparency of the XGBoost model.

In addition, to assess each risk factor's contribution, two complementary analyses were conducted. First, a leave‐one‐feature‐out (LOFO) analysis was performed by omitting one feature at a time from the full predictor set, followed by retraining the XGBoost model using the NCV and seed averaging protocols. The resulting change in ROC AUC and PR AUC relative to the full model provided insights into the strength of each feature's contribution. Second, individual models were built using only one risk factor at a time. The ROC AUC and PR AUC scores obtained from these single‐feature models reflect the standalone predictive power of each feature. These analyses allowed for a robust and supplementary comparison of the impacts of individual risk factors on dementia conversion prediction.

To further ensure robustness and stability of the findings, repeated experiments with seed averaging were incorporated in all the analysis. Seed averaging involves training the model multiple times using different random seeds to mitigate the impact of randomness. The performance metrics (accuracy, sensitivity, specificity, ROC AUC, and PR AUC) and SHAP values from each run were then averaged, ensuring a stable estimate of the model's true performance and enhancing the assessment of its generalization capability.^31^, ^32^

### 2.5 Hypothesis‐driven approach

A comprehensive hypothesis‐driven approach to evaluate the impact of risk factors on dementia conversion was conducted. Baseline characteristics were summarized using descriptive statistics, with continuous variables reported as mean (SD) and categorical variables presented as frequencies and percentages.

Survival analysis utilized Kaplan–Meier curves and Cox proportional hazards regression. Participants were stratified into quartiles (Q1–Q4) based on risk factor count, with Q1 and Q2 classified as “low risk” and Q3 and Q4 as “high risk.” Kaplan–Meier curves visualized time‐to‐first dementia diagnosis, estimating dementia‐free probability across risk groups, with log‐rank tests comparing survival distributions.

Cox models quantified the impact of multiple risk factors, estimating hazard ratios (HRs) with 95% confidence intervals (CIs) and *p*‐values. The overall significance of the model was assessed using the likelihood ratio test. Proportional hazards assumptions were tested using Schoenfeld residuals; however, considering our large sample size, even small violations of this assumption may be detected.^33^ For variables where minor violations are found, those will be interpreted as an average effect over time.^34^

The integrated approach combined ML techniques with traditional survival analysis to provide a comprehensive evaluation of dementia risk factors, enabling a nuanced interpretation of potential cognitive decline predictors

### 2.6 Computational environment

The analysis was conducted using Jupyter Lab with Python 3.11.7 on a Windows 10 workstation equipped with 16 cores and 16 GB RAM. All Python libraries, their versions, and purposes are detailed in Table S2 to ensure reproducibility.

## 3 RESULTS

### 3.1 Characteristics of study participants

A total of 9606 individuals were included in the analysis. Of those, 877 (9%) converted to dementia and 8729 (91%) remained cognitively unimpaired (Table 1). Most of the population comprised elderly (mean age ± SD, 70.1 ± 9.9 years), females (65%), and White individuals (82%) with a mean follow‐up of 6.0 (SD 4.2) years. Compared to individuals who remained cognitively unimpaired at follow‐up, those in the group that converted to dementia presented increased age at baseline, Clinical Dementia Rating Sum of Boxes (CDR‐SOB) scale score, follow‐up time, GDS, lower BMI, and less education (*p* < 0.001 for all, Table 1). The correlation matrix revealed that no variables present in the dataset have a correlation coefficient greater than 0.3 (Figure S2 **)**.

**TABLE 1:** Baseline demographic and clinical characteristics of the study cohort, divided by dementia conversion status.

Characteristic	Stable group (*n* = 8729)	Converted group (*n* = 877)	Total population (*n* = 9606)	*p*‐value
Age, mean (SD)	69.44 ± 9.75	76.59 ± 9.10	70.09 ± 9.91	**<0.001**
Education, years, mean (SD)	16.08 ± 2.80	15.50 ± 2.91	16.02 ± 2.81	**<0.001**
BMI, mean (SD)	27.42 ± 5.37	26.23 ± 4.34	27.31 ± 5.30	**<0.001**
Geriatric Depression Scale, mean (SD)	1.11 ± 1.74	1.41 ± 1.96	1.14 ± 1.77	**<0.001**
Follow‐up time, years, mean (SD)	5.75 ± 4.12	8.87 ± 4.05	6.03 ± 4.21	**<0.001**
CDR‐SOB, mean (SD)	0.07 ± 0.27	0.18 ± 0.50	0.08 ± 0.30	**<0.001**
Female, n (%)	5699 (65.3%)	559 (63.7%)	6258 (65.1%)	0.379
Race, n (%)				**<0.001**
White	7133 (81.7%)	780 (88.9%)	7913 (82.4%)	
Black or African American	1238 (14.2%)	83 (9.5%)	1321 (13.8%)	
Asian	229 (2.6%)	10 (1.1%)	239 (2.5%)	
American Indian or Alaska Native	54 (0.6%)	0 (0.0%)	54 (0.6%)	
Other	42 (0.5%)	2 (0.2%)	44 (0.5%)	
Native Hawaiian or Other Pacific Islander	6 (0.1%)	0 (0.0%)	6 (0.1%)	
Hearing loss, n (%)	808 (9.3%)	75 (8.6%)	883 (9.2%)	0.531
Hyperlipidemia, n (%)	4333 (49.6%)	423 (48.2%)	4756 (49.5%)	0.448
TBI, n (%)	990 (11.3%)	86 (9.8%)	1076 (11.2%)	0.187
Diabetes, n (%)	906 (10.4%)	88 (10.0%)	994 (10.3%)	0.794
Smoking, n (%)	3686 (42.2%)	388 (44.2%)	4074 (42.4%)	0.265
Hypertension, n (%)	3.949 (45.2%)	444 (50.6%)	4.393 (45.7%)	**0.003**
Alcohol abuse, n (%)	273 (3.1%)	24 (2.7%)	297 (3.1%)	0.593
Visual loss, n (%)	341 (3.9%)	42 (4.8%)	383 (4.0%)	0.237

Abbreviations: BMI, body mass index; CDR‐SOB, Clinical Dementia Rating Sum of Boxes; SD, standard deviation; TBI, traumatic brain injury.

Bolded values indicate statistically significant predictors (*p* < 0.05).

### 3.2 Data‐driven results

**FIGURE 2:**
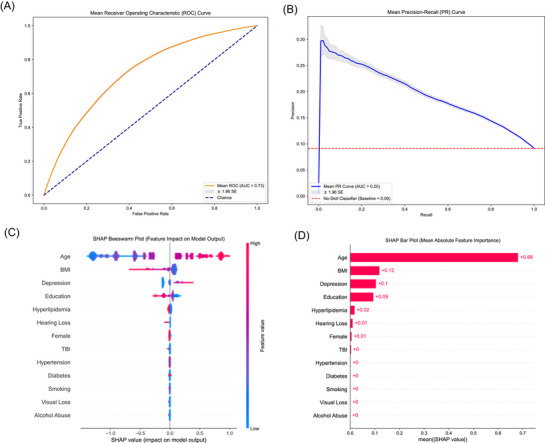
Predictive performance and the contribution of each risk factor of the data‐driven approach: (A) The mean ROC curve across all seeds, (B) the mean PR curve, and (C) the SHAP Beeswarm plot illustrates the impact of each feature on the model's output for every participant. Each dot represents a participant; its position on the *x*‐axis indicates the SHAP value (positive values increase the prediction of dementia), and its color represents the feature's value (red for high, blue for low). (D) The SHAP bar plot ranks features by their overall importance, calculated as the mean absolute SHAP value across all participants. AUC, area under the curve; BMI, body mass index; PR, precision recall; ROC, receiver‐operating characteristic; SHAP, SHapley Additive exPlanations; TBI, traumatic brain injury.

The outcome of the data‐driven approach yielded a mean ROC AUC of 0.732 (95% CI: 0.729–0.734) and a mean PR AUC of 0.208 (95% CI: 0.205–0.210), indicating moderate discrimination performance, as presented in Figure 2A,B. The narrow 95% CI reflects consistency across seeds. At the optimal threshold determined by Youden's J Statistic, the model achieved a mean accuracy of 0.643 (95% CI: 0.634–0.652), a mean sensitivity of 0.702 (95% CI: 0.688–0.714), and a mean specificity of 0.637 (95% CI: 0.626–0.648). These metrics reflect a balanced trade‐off between sensitivity and specificity. The inner loop metrics of training obtained were a mean ROC AUC of 0.684 (95% CI: 0.680–689) and a mean PR AUC of 0.162 (95% CI: 0.160–164); compared with the validation set in the inner loop obtained, there is no sign of overfitting, with mean values of ROC AUC 0.666 (95% CI: 0.662–0.669) and mean PR AUC 0.152 (95% CI: 0.150–0.153).

Age emerged as the most influential feature in the SHAP analysis (Figure 2C), with the highest mean absolute SHAP value of 0.68. The summary plot (Figure 2C) indicated a strong and consistent impact of age on the model predictions, with higher age values contributing positively to the conversion to dementia. Other notable contributors included BMI, depression, and education, (mean SHAP values = 0.12, 0.10, and 0.09, respectively) (Figure 2D). These features had a moderate but consistent impact on model predictions. In detail, a higher BMI, lower rates of depression, and higher education were associated with a lower likelihood of conversion to dementia. Other features (TBI, hyperlipidemia, diabetes, sex, alcohol abuse, hearing loss, visual loss, hypertension, and smoking) had a much lower SHAP value, indicating a minimal influence on model's predictions. This finding aligns with our supplementary analyses, where age, BMI, depression, and education were consistently identified as the most influential features (Figure S3).‘’

### 3.3 Hypothesis‐driven results

**FIGURE 3:**
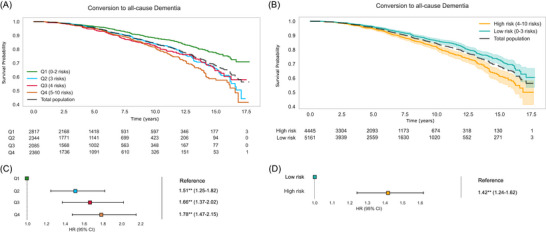
Survival analysis for time to dementia diagnosis. (A) The Kaplan–Meier curve displays the dementia‐free probability for the total study population over a follow‐up of up to 17.5 years, with the number of participants at risk below the curves, according to quartiles (Q1–Q4) based on their total count of risk factors at baseline. (B) The Kaplan–Meier curves show different rates of conversion to all‐cause dementia according to high‐risk (4–10 risk factors) versus low‐risk groups (0–3 risk factors). (C) The hazard ratios (HRs) and 95% CIs from a Cox proportional hazards model for dementia conversion, using Q1 as reference. (D) A similar forest plot shows the HRs comparing participants in the low‐risk group (0–3 risk factors) versus the high‐risk group (4–10 risk factors), using the low‐risk group as the reference group. CI, confidence interval; HR, hazard ratio.

The cumulative dementia‐free probability curve for the entire study population demonstrates a gradual decline in the dementia‐free probability over the follow‐up period (Figure 3A). The number at risk decreases steadily, accompanied by an increasing number of censored observations and events over time, as expected in real‐world cohorts. At 17.5 years of follow‐up, 1052 individuals converted to dementia (Figure 3A).

Risk factor stratification was performed to investigate the potential heterogeneity in conversion to dementia (Figure 3A). This subgroup analysis revealed distinct cumulative dementia‐free probabilities. Participants classified as low risk—Q1 and Q2—exhibited a higher cumulative dementia‐free probability across the timeline compared to those classified as high risk—Q3 and Q4 (Figure 3B). The curves for low‐ and high‐risk individuals began to diverge significantly after ∼5 years of follow‐up, indicating an increased rate of dementia diagnosis among individuals in the higher‐risk quartiles. These results suggest that higher risk factor scores are associated with a greater likelihood of earlier dementia diagnosis (Figure 3C,D), emphasizing the impact of the investigated factors on conversion. An individual plot for each specific risk factor is available in Figure S4.

**FIGURE 4:**
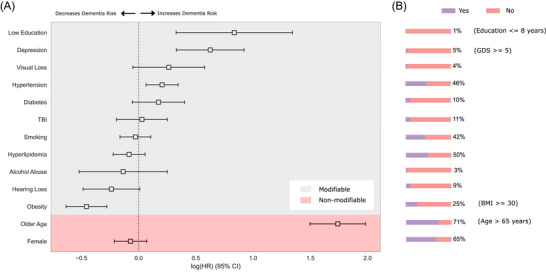
Forest plot generated from the hypothesis‐driven Cox proportional hazards model. (A) Each of the 13 risk factors evaluated simultaneously in the multivariable model. (B) The bar charts illustrate the prevalence (percentage of positive cases) of each modifiable risk factor within the entire study cohort (*N* = 9606) at baseline. For age, the chart shows the percentage of participants equal or older than 65 years at baseline. BMI, body mass index; GDS, Geriatric Depression Scale; HR, hazard ratio; TBI, traumatic brain injury.

The Cox models were employed to quantify the relationship between the investigated risk factors and the time to the first diagnosis of dementia (Figure 4). The HR and prevalence of each modifiable risk factor differed substantially, as seen in Table 2. The model demonstrated moderate predictive capability, with a concordance index (C‐index) of 0.68, indicating good discriminatory power. The log‐likelihood ratio test was highly significant (*p* < 0.005, –log2(p) = 390.20), confirming the model's overall fit.

**TABLE 2:** Prevalence in the whole sample and multivariable hazard ratios for the 13 risk factors of dementia conversion.

Risk factor	Prevalence (%)	HR (95% CI)	*p*‐values
Older age	71%	5.69 (4.47–7.25)	**<0.005**
Low education	1%	2.31 (1.39–3.83)	**<0.005**
Depression	5%	1.87 (1.39–2.51)	**<0.005**
Hypertension	46%	1.23 (1.07–1.41)	**<0.005**
Visual loss	4%	1.30 (0.95–1.78)	0.10
Diabetes	10%	1.19 (0.95–1.49)	0.14
TBI	11%	1.03 (0.82–1.28)	0.80
Smoking	42%	0.97 (0.85–1.11)	0.68
Female	65%	0.93 (0.81–1.07)	0.32
Hyperlipidemia	50%	0.92 (0.80–1.06)	0.24
Alcohol abuse	3%	0.87 (0.59–1.28)	0.49
Hearing loss	9%	0.79 (0.62–1.01)	0.06
Obesity	25%	0.63 (0.53–0.76)	**<0.005**

Abbreviations: CI, confidence interval; HR, hazard ratio; TBI, traumatic brain injury.

Bolded values indicate statistically significant predictors (*p* < 0.05).

In this model, older age increased the HR of dementia diagnosis by 469% (HR: 5.69, 95% CI: 4.47–7.25, *p* < 0.005), highlighting age as a major determinant of dementia risk. Less educational attainment was associated with a 131% increase in HR of dementia (HR: 2.31, 95% CI: 1.39–3.83, *p* < 0.005). Depression had an 87% increase in HR of dementia (HR: 1.87, 95% CI: 1.39–2.51, *p* < 0.005), and hypertension a 23% increase (HR: 1.23, 95% CI: 1.07–1.41, *p* < 0.005). Regarding protective factors, obesity was associated with a 37% decrease in HR (HR: 0.63, 95% CI: 0.53–0.76, *p* < 0.005). Older age, hyperlipidemia, and hearing loss appeared as minor violations in the proportional hazards assumption check, so should be interpreted as an average effect over time. The remaining features all passed the proportional hazards assumption check.

## 4 DISCUSSION

This study stratified multiple risk factors of dementia simultaneously, using a hypothesis‐ and data‐driven approaches in a large real‐world cohort. Our findings highlight the importance of evaluating the combination of risk factors to predicting dementia conversion, instead of assessing risk factors separately. More specifically, both of our models identified that age, depression, education, and BMI are the most important predictors of conversion to dementia. These findings may have a clinical impact, since they can be assessed during a single medical consultation.

In this study, investigating multiple risk factors simultaneously provided a thorough understanding of their ability to predict the conversion to dementia. Our findings underscore that, among the 13 risk factors evaluated (11 modifiable and 2 non‐modifiable), only 4 risk factors were consistently associated with conversion in hypothesis‐ and data‐driven models. Previous studies have consistently demonstrated the effect of each risk factor independently predicting the risk of dementia. However, multimorbidity is highly prevalent in older adults, and the analysis of risk factors separately is insufficient to understand the cumulative interaction between different conditions in dementia risk.^35^ Collinearity among risk factors may under‐ or overestimate their impact in real‐world memory clinics.^4^ More specifically, the majority of older adults with hypertension may also present dyslipidemia,^36^ although these factors were not usually evaluated in previous risk factor analyses. Moreover, the cumulative effect of different factors, such as depression and cardiovascular risk factors, may synergistically increase the risk of dementia.^37^ This real‐world longitudinal data from the NACC provides a unique possibility to evaluate the mutual effect of multiple risk factors and their mutual influence in developing dementia.

Age, depression, and less education were consistently associated with conversion to dementia in our models. Age was the most impactful risk factor of dementia identified in hypothesis and data‐driven models. First, age remains the strongest non‐modifiable factor associated with cognitive decline across multiple studies.^38^, ^39^ Second, depression has been repeatedly identified as a risk factor for dementia in a complex interplay with cause–consequence effects.^40^ More specifically, depression and dementia may share common genetic predisposition,^41^ depressive symptoms are increasingly associated with prodromal phases of AD,^42^ and dementia may also cause major depression.^43^ Of note, analyzing risk factors simultaneously demonstrated that depression had one of the highest impacts on dementia conversion, corroborating previous data.^4^ Depressive symptoms in the elderly are increasingly identified as potential prodromal symptoms of AD, whose relationship remains unclear.^42^ Third, low education is a significant modifiable early life risk factor for dementia. This association is observed globally, including in low‐ and middle‐income countries, where low education often represents broader socioeconomic disadvantage.^44^, ^45^ Jointly, age, depression, and less education present a potential mutual negative effect on cognition. Recent studies have demonstrated that depression in midlife increases the risk of dementia, whereas in older adults the association between depression and dementia was less strong.^46^ Therefore, evaluating risk factors individually may not capture the complex interplay between them.

Substance use, specifically smoking and excessive alcohol use, were consistently associated with a higher risk of dementia in previous data.^4^, ^47^, ^48^ However, they were not associated with conversion in this data‐driven and hypothesis‐driven analyses. A potential explanation for this difference may be associated with the complementary effect among risk factors that was not observed when evaluating risk factors individually, as mentioned for major cardiovascular events.^49^ In addition, previous meta‐analyses^47^, ^48^ that identified increased risk for dementia in substance use included heterogeneous methods of evaluating alcohol use, whereas the current analysis included a single method for either alcohol and smoking.

Surprisingly, BMI reduced the risk of conversion to dementia in our analysis. Although the risk factor was associated with an increased risk of dementia,^4^ conflicting data were published previously. More specifically, higher BMI plays a paradoxical role in the risk of dementia, varying according to diagnosis at baseline and the follow‐up period.^50^ In addition, a meta‐analysis presented that underweight individuals also presented an increased risk of dementia, and the authors discussed that older adults might potentially lose weight before conversion to dementia.^51^ Remarkably, in a longitudinal study including 1.3 million adults from three continents, higher BMI was associated with increased dementia risk when measured >20 years before dementia diagnosis—but high BMI was protective when assessed <10 years before diagnosis of dementia.^50^ This raises the possibility of confounding, where the protective effect of BMI could be an artifact of age‐related weight loss. However, the correlation between age and BMI at baseline was very weak (*r* = –0.08) in our cohort, making it unlikely that age is the sole explanation for this finding. The observed protective effect of a higher BMI, even after accounting for related factors like diabetes and hypertension, highlights a key strength of our multivariable approach. Including correlated risk factors within the same model allows for the estimation of the independent contribution of each variable. The persistence of BMI's protective effect suggests that it is not merely an artifact of its correlation with other cardiometabolic conditions, reinforcing the value of a simultaneous, multi‐factor analysis to uncover the complex and independent roles of each risk factor.

A key strength of our dual‐method approach relies on its ability to robustly handle variables with low prevalence like educational attainment. More specifically, Cox models reflect uncertainty through wider CIs, whereas XGBoost identifies feature importance through outcome separation. The consistent identification of low education as significant across both models underscores our analytical strategy's robustness. This study offers immediate clinical utility, as all variables can be collected in a single consultation, enabling rapid risk assessment. Our approach using baseline risk factors to predict longitudinal outcomes intentionally mirrors real‐world clinical scenarios, where age strongly influences dementia prediction as a primary risk factor rather than a model limitation.

This study presents many limitations. It lacks external validation in independent cohorts and national representativeness, consisting primarily of White volunteers from specialized research centers. In addition, three Lancet Commission modifiable risk factors (air pollution, physical exercise, and social isolation) were unavailable. Our methodological limitations include using binary risk factor classification for some variables, not accounting for risk factor onset timing or treatment effects. Incomplete participant tracking and censoring bias in the NACC database further constrain result interpretation. Despite these limitations, our study provides a valuable methodological framework for comparing analytical approaches to identify population‐specific dementia risk factors. Further studies with representative populations from the United States, from the Global South, and more diverse cohorts should be conducted to corroborate or not these findings. Moreover, future study designs may evaluate the cumulative time of risk factors and their impact on cognitive outcomes, and may include more granular variables (i.e., blood pressure and plasma glucose level).

In summary, this real‐world longitudinal analysis stratified the most impactful risk factors in clinical practice. Age, depression, and low education were major risk factors for conversion to dementia in both data‐ and hypothesis‐driven models, whereas higher BMI reduced the risk. Real‐world analyses enable the investigation of multimorbidity effects of risk factors simultaneously, whereas diverse analytical models capture the complexity of their interactions.

## CONFLICT OF INTEREST STATEMENT

E.R.Z. has served in the scientific advisory board of Nintx, Novo Nordisk, and Masima. He is also a co‐founder and a minority shareholder at Masima. W.V.B. and M.A.B. are co‐founders and minority shareholders at Masima. D.A., R.C.B., and J.P.F. have nothing to disclose. Any author disclosures are available in the supporting information.

## CONSENT STATEMENT

At each Alzheimer's Disease Research Center, all participants or their caregivers provided written informed consent for participation in the study, a process that was approved by the respective institutional review boards at each site.

## Supporting information



Supporting information

Supporting information
