# Supplementary material for: Stratifying dementia risk factors: A prediction model and hypothesis‐driven analysis
Source: Alzheimers Dement. 2025 Oct 28;21(10):e70870. doi: 10.1002/alz.70870 (PMC12568388; doi:10.1002/alz.70870)
Supplement: Supplementary file 1 — Supporting information [file ALZ-21-e70870-s002.docx]

**Running title**: Stratifying dementia risk factors: A prediction model and hypothesis-driven analysis

**Authors:**

Daniel Arnold^a^

Rodrigo C Barros^b^

João Pedro Ferrari-Souza^c^

Marco Antonio de Bastiani^a^

Eduardo R Zimmer^a,c,d,f,g^

Wyllians Vendramini Borelli^a,c,h,i^

**Affiliations:**

^a^ Graduate Program in Biological Sciences: Pharmacology and Therapeutics, Universidade Federal do Rio Grande do Sul (UFRGS), Porto Alegre, RS, Brazil.

^b^ Graduate Program in Computer Science (PPGCC), School of Technology, Pontifical Catholic University of Rio Grande do Sul (PUCRS), Porto Alegre, RS, Brazil.

^c^ Graduate Program in Biological Sciences: Biochemistry, Universidade Federal do Rio Grande do Sul (UFRGS), Porto Alegre, RS, Brazil.

^d^ Department of Pharmacology, Universidade Federal do Rio Grande do Sul (UFRGS), Porto Alegre, RS, Brazil.

^f^  The McGill Centre for Studies in Aging, McGill University

^g^ Brain Institute of Rio Grande do Sul, Pontifical Catholic University of Rio Grande do Sul (PUCRS), Porto Alegre, RS, Brazil.

h Department of Morphological Sciences, Universidade Federal do Rio Grande do Sul (UFRGS), Brazil

^i^ Memory Center at the Hospital Moinhos de Vento, Brazil

**Corresponding author:**

Wyllians Vendramini Borelli, MD, PhD

500 Sarmento Leite St, Porto Alegre, Brazil

Zip code 90050-170

[wyllians.borelli@ufrgs.br](mailto:wyllians.borelli@ufrgs.br)

**Supplementary Figure 1.** Flowchart illustrating the participant selection process from the National Alzheimer's Coordinating Center (NACC) dataset.


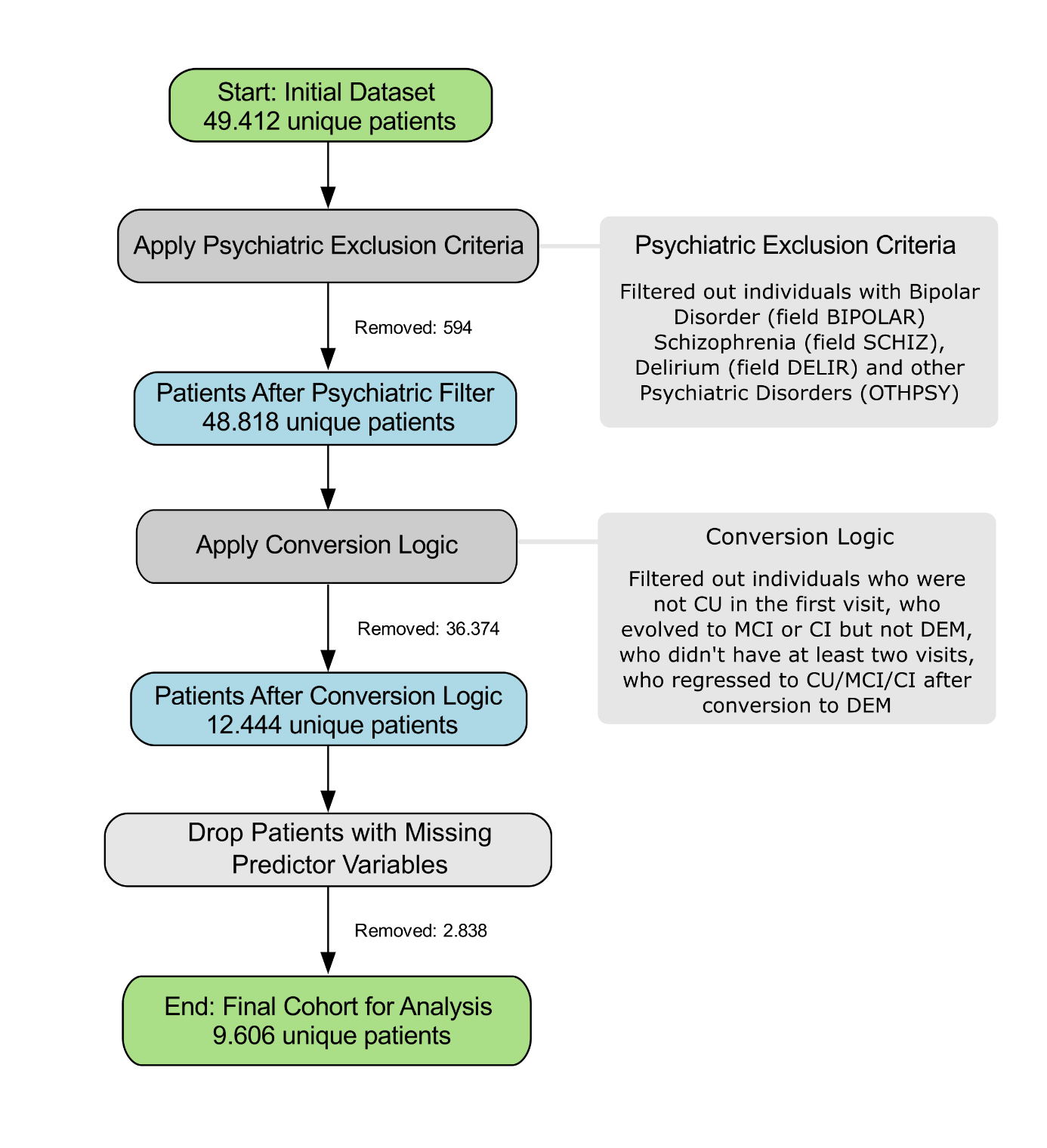


This flowchart details the sequential application of exclusion criteria to arrive at the final analytical cohort. Starting with 49,412 unique participants from the NACC database, individuals were removed based on: (1) pre-existing psychiatric conditions (e.g., Bipolar Disorder, Schizophrenia), (2) conversion logic (e.g., not cognitively unimpaired at baseline, not converting to dementia, or having fewer than two visits), and (3) missing data for any of the 13 predictor variables. This process resulted in a final cohort of 9,606 participants for the analysis.

**Supplementary Figure 2.** Heatmap of the Pearson correlation matrix for the 13 risk factors included in the analysis.

**
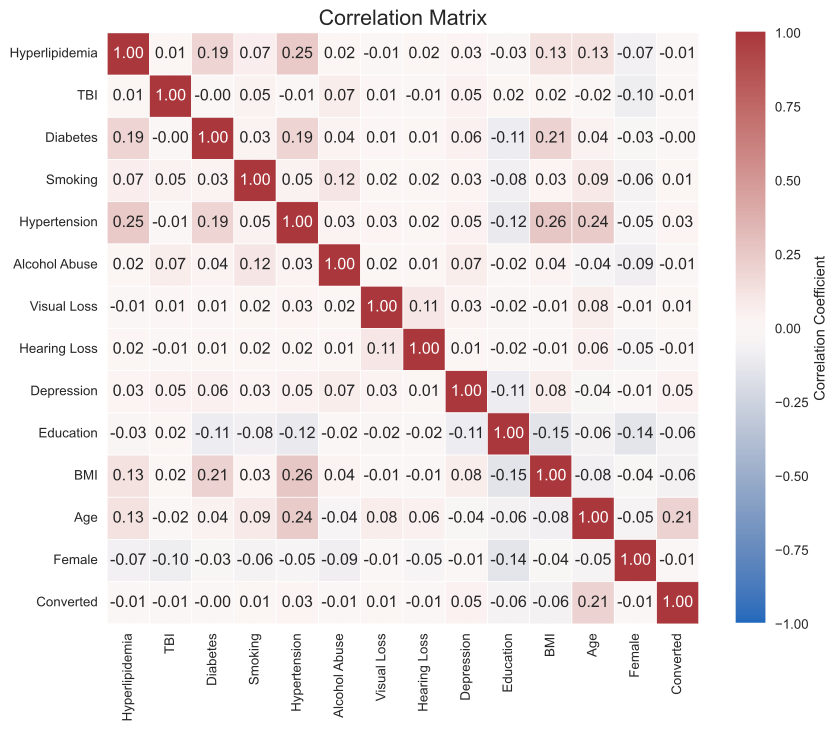
**

The figure displays the Pearson correlation coefficients between all pairs of the 13 risk factors used in the models. The color and intensity of each square represent the direction and strength of the correlation, with warmer colors (e.g., red) indicating a positive correlation and cooler colors (e.g., blue) indicating a negative correlation. The number in each square is the exact correlation coefficient. This analysis was performed to assess multicollinearity among the predictors. As no strong correlations (e.g., > 0.7 or < -0.7) were observed, all 13 features were retained for the modeling.

**Supplementary Figure 3.** Evaluation of the predictive power of individual risk factors using leave-one-feature-out importance and single-feature models.


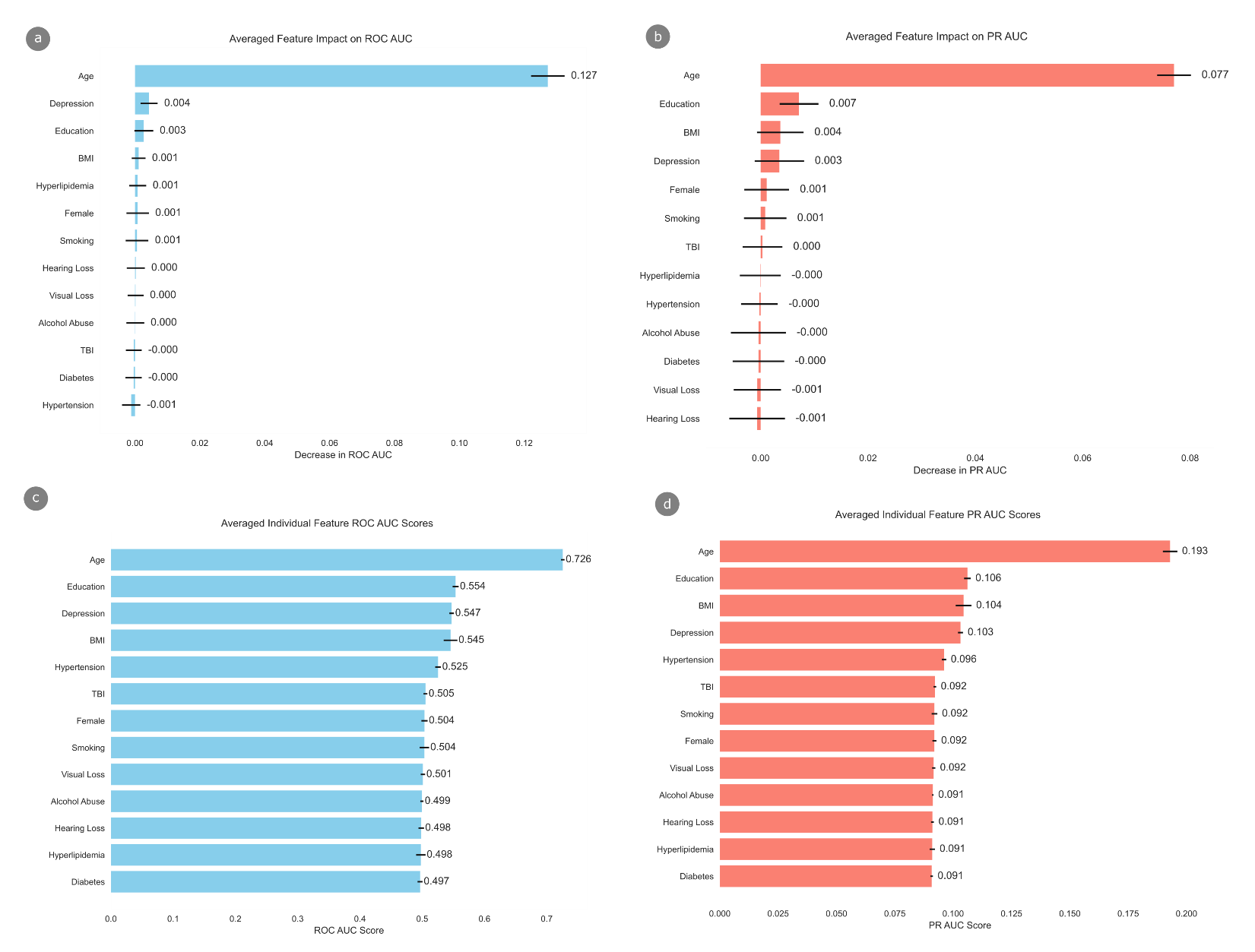
This figure assesses the contribution of each risk factor to the model's performance. (a, b) Leave-one-feature-out importance for the full XGBoost model. The bars show the average decrease in the model's Area Under the Curve (AUC) for the Receiver Operating Characteristic (ROC) curve (a) and the Precision-Recall (PR) curve (b) when a single feature's is removed. A larger decrease indicates a more important feature. (c, d) Performance of individual models trained on only one feature at a time. The bars represent the ROC AUC (c) and PR AUC (d) for each single-feature model, providing a direct measure of each feature's standalone predictive capability. Abbreviations: AUC - Area Under the Curve; ROC - Receiver Operating Characteristic; PR - Precision-Recall; BMI - Body Mass Index.

**Supplementary Figure 4.** Univariate Kaplan-Meier survival analyses for dementia-free probability, stratified by the presence or absence of each individual risk factor.


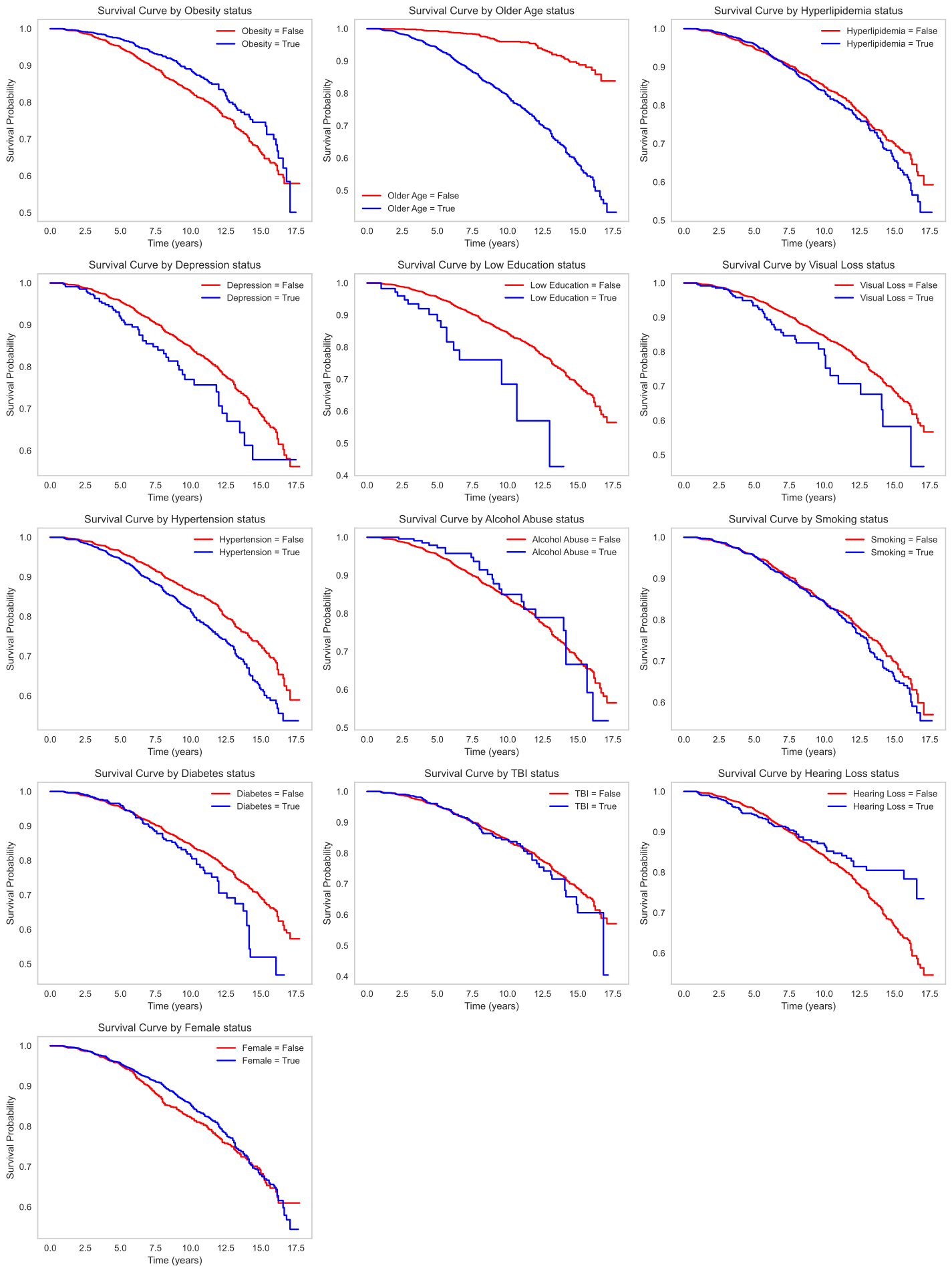


Each plot in this figure displays a Kaplan-Meier curve showing the probability of remaining dementia-free over the follow-up period. In each plot, the cohort is stratified into two groups based on the status of a single risk factor at baseline. These univariate analyses visualize the unadjusted association between each individual risk factor and the time to dementia diagnosis. The x-axis represents time in years, and the y-axis represents the survival probability.

**Supplementary Table 1**. Search space for hyperparameter optimization of the XGBoost model.

| Hyperparameter | Search Range | Purpose |
| --- | --- | --- |
| n_estimators | Integer from 50 to 300 | The total number of trees to build in the ensemble. More trees can improve performance but increase computation time. |
| max_depth | Integer from 2 to 10 | The maximum depth of an individual tree. Deeper trees can capture more complex patterns but are more prone to overfitting. |
| learning_rate | Float from 0.01 to 0.3 (log scale) | Scales the contribution of each tree. A smaller value requires more trees but often leads to a more robust model. |
| subsample | Float from 0.3 to 1.0 | The fraction of training data instances (rows) to be randomly sampled for building each tree. Helps prevent overfitting. |
| colsample_bytree | Float from 0.3 to 1.0 | The fraction of features (columns) to be randomly sampled when constructing each tree. |
| gamma | Float from 0 to 5 | Minimum loss reduction required to make a split. A larger value makes the algorithm more conservative. |
| reg_alpha | Float from 10−8 to 10.0 (log scale) | L1 regularization term on weights. It encourages sparsity and can help with feature selection by shrinking some weights to zero. |
| reg_lambda | Float from 10−8 to 10.0 (log scale) | L2 regularization term on weights. It penalizes large weights, making the model simpler and less prone to overfitting. |
| scale_pos_weight | Float from 1 to 10 | A weight to balance the positive and negative classes, which is especially useful for imbalanced datasets. |
| min_child_weight | Integer from 1 to 10 | The minimum sum of instance weight (hessian) needed in a child node. It helps control overfitting by preventing splits on nodes with low observation counts. |

This table details the range of values explored for each hyperparameter of the XGBoost algorithm during the model optimization phase. The optimization was conducted within the inner loop of the nested cross-validation procedure using the Optuna framework. The final model utilized the combination of hyperparameters from this search space that yielded the best performance on the validation sets. This systematic approach ensures that the final model is well-tuned to the dataset, balancing predictive accuracy with complexity to avoid overfitting.

**Supplementary Table 2.** List of Python libraries and software versions used in the analysis.

| Library | Version | Purpose in this Analysis |
| --- | --- | --- |
| collections | Standard Library | Provides high-performance container datatypes beyond the standard dict, list, set, and tuple. |
| copy | Standard Library | Used for creating shallow and deep copies of objects. |
| dill | 0.3.6 | An advanced serialization library for saving and loading the entire Python session. |
| graphviz | 0.21 | A tool for creating graph visualizations, used here to generate the data filtering flowchart. |
| hyperopt | 0.2.7 | A library for distributed asynchronous hyperparameter optimization. |
| joblib | 1.4.2 | Used for simple parallel computing, particularly for running cross-validation tasks on multiple cores. |
| lifelines | 0.30.0 | A complete survival analysis library, used for Kaplan-Meier curves and Cox proportional hazards models. |
| logging | Standard Library | Logs events and messages from the script for debugging and monitoring. |
| math | Standard Library | Provides access to basic mathematical functions. |
| matplotlib | 3.9.2 | A primary library for creating static, animated, and interactive visualizations and plots. |
| missingno | 0.5.2 | Provides a small toolset of flexible and easy-to-use missing data visualizations. |
| multiprocessing | Standard Library | Enables running processes in parallel, improving performance on multi-core systems. |
| numpy | 1.26.4 | The fundamental package for numerical computing, supporting large, multi-dimensional arrays and matrices. |
| optuna | 4.0.0 | An automatic hyperparameter optimization framework used to find the best model parameters. |
| os | Standard Library | Interacts with the operating system, such as for file path manipulation. |
| pandas | 2.2.3 | Essential for data manipulation and analysis, offering structures like the DataFrame for tabular data. |
| random | Standard Library | Generates random numbers, used for seeding to ensure reproducibility. |
| re | Standard Library | Provides regular expression operations for text pattern matching. |
| scipy | 1.10.1 | A core library for scientific and technical computing, used here for statistical functions like sem and t-test. |
| seaborn | 0.13.2 | A high-level data visualization library based on matplotlib that provides attractive statistical graphics. |
| shap | 0.46.0 | Explains the output of machine learning models, providing insights into feature importance and impact. |
| sklearn | 1.2.2 | The go-to machine learning library, providing tools for model selection, metrics, preprocessing, and more. |
| subprocess | Standard Library | Used to spawn new processes, for example, to install packages from within a script. |
| sweetviz | 2.3.1 | A library for automated Exploratory Data Analysis (EDA), creating beautiful, high-density visualizations. |
| sys | Standard Library | Provides access to system-specific parameters and functions. |
| warnings | Standard Library | Controls how warning messages are handled and displayed. |
| xgboost | 2.0.3 | An optimized distributed gradient boosting library designed for efficiency, flexibility, and portability. |
| yellowbrick | 1.5 | A suite of visual analysis and diagnostic tools to help select the best machine learning model. |

This table provides a comprehensive list of the key Python libraries and their specific versions used for all data processing, statistical analysis, machine learning modeling, and figure generation. The purpose of each library within the analytical pipeline is also described.
